# Deep learning-based prediction of one-year mortality in Finland is an accurate but unfair aging marker

**DOI:** 10.1038/s43587-024-00657-5

**Published:** 2024-06-24

**Authors:** Andrius Vabalas, Tuomo Hartonen, Pekka Vartiainen, Sakari Jukarainen, Essi Viippola, Rodosthenis S. Rodosthenous, Aoxing Liu, Sara Hägg, Markus Perola, Andrea Ganna

**Affiliations:** 1 Institute for Molecular Medicine Finland (FIMM), HiLIFE, University of Helsinki, Helsinki, Finland; 2 Pediatric Research Center, Helsinki University Hospital and University of Helsinki, Helsinki, Finland; 3 Broad Institute of MIT and Harvard, Cambridge, MA USA; 4 Department of Medical Epidemiology and Biostatistics, Karolinska Institutet, Stockholm, Sweden; 5 The Finnish Institute for Health and Welfare, Helsinki, Finland; 6 Analytic and Translational Genetics Unit, Massachusetts General Hospital, Boston, MA USA

**Keywords:** Predictive markers, Predictive markers, Ageing

## Abstract

Short-term mortality risk, which is indicative of individual frailty, serves as a marker for aging. Previous age clocks focused on predicting either chronological age or longer-term mortality. Aging clocks predicting short-term mortality are lacking and their algorithmic fairness remains unexamined. We developed a deep learning model to predict 1-year mortality using nationwide longitudinal data from the Finnish population (FinRegistry; *n* = 5.4 million), incorporating more than 8,000 features spanning up to 50 years. We achieved an area under the curve (AUC) of 0.944, outperforming a baseline model that included only age and sex (AUC = 0.897). The model generalized well to different causes of death (AUC > 0.800 for 45 of 50 causes), including coronavirus disease 2019, which was absent in the training data. Performance varied among demographics, with young females exhibiting the best and older males the worst results. Extensive prediction fairness analyses highlighted disparities among disadvantaged groups, posing challenges to equitable integration into public health interventions. Our model accurately identified short-term mortality risk, potentially serving as a population-wide aging marker.

## Abstract (web summary)

Here the authors show that deep learning accurately predicts one-year mortality using nationwide Finnish data. Despite robust performance and potential as an aging marker, fairness analyses reveal prediction disparities, urging cautious integration into public health.

## Main

Understanding the mechanisms leading to death and the sources of increased biological heterogeneity in old age remains a central question in aging research^1^. Measuring the state of aging of an individual (that is, their biological age) is a crucial step to address this question.

Molecular aging clocks serve as the primary means to measure biological age. They were initially developed to forecast chronological age by leveraging various omics data, demonstrating notable accuracy for this purpose^2–8^. However, their capacity to predict mortality beyond chronological age—a more pertinent task in understanding aging mechanisms—has shown modest outcomes. A subsequent generation of molecular aging clocks has been specifically trained to predict biological age using biomarker and mortality data. Although advancements have been made in predictions, more work is needed^9,10^. Recently, a multimodal score trained on UK Biobank data encompassing not only omics but also demographic, medical and lifestyle information, has achieved commendable predictive accuracy for 5-year and 10-year mortality^11^.

Beyond the utility of aging clocks in explaining the variability in aging trajectory, determining life expectancy and mortality risk remain fundamental for public health, medical research and policy-making^12,13^. The accurate identification of individuals at risk of short-term death is pivotal for planning risk-reducing interventions. Short-term mortality prediction holds substantial value in enhancing the quality of end-of-life care while concurrently optimizing healthcare resource allocation to minimize costs^14^.

Recent advances in machine learning, coupled with the wider availability of digitized medical and socioeconomic information at a population level, paved the way for the development of algorithms that can predict patients’ future health trajectories and aid medical decision-making^15,16^. Deep learning (DL) models can leverage massive amounts of data, requiring minimal preprocessing or feature engineering. A clear advantage of DL models is the possibility to analyze an individual’s longitudinal history, considering time intervals elapsed between different events, including medical encounters, as well as socioeconomic information.

Unlike traditional statistical methods, DL is often viewed as a ‘black box’, a term meaning that its decisions are difficult to interpret. While existing explainability methods can provide insights into which attributes are important at the level of an individual, they do not facilitate the understanding of differences in predictions across groups of individuals^17^. Understanding how model performance varies across different groups is especially important when considering issues of fairness. Fair algorithms should not exhibit bias or preference toward any individual or group based on inherent or acquired attributes^18^. There have been instances where DL algorithms are unfair^19^, particularly when they perform poorly for socially disadvantaged individuals, who may face higher barriers to accessing healthcare, resulting in more missing data and measurement errors that ultimately skew the predictions^20,21^.

For instance, Fong et al.^22^ found that a model predicting hospital readmissions achieved much higher prediction accuracy among self-reported White individuals compared to other ethnic groups. Similarly, Meng et al.^23^ identified disparities in the frequency of mechanical ventilation interventions across different ethnicities, sexes and ages, leading to differences in prediction accuracy across groups. Chen et al.^24^ found that prediction models performed worse for males compared to females and among individuals with public, rather than private, health insurance.

Our study aimed to accurately predict 1-year mortality for every Finnish resident by using comprehensive, nationwide, multi-category information and to evaluate how prediction accuracy varies within different groups defined according to health, geographical location and socioeconomic characteristics. To achieve this objective, we developed a state-of-the-art DL model.

In contrast to previously developed aging clocks^2–11^ and studies focusing on mortality prediction using electronic health record^25^, environmental and lifestyle factor^26^ and biomarker data^27^, we introduced three key innovations. First, we used a substantially larger sample size by including the entire Finnish population, thereby mitigating ascertainment bias. Second, we leveraged an unprecedented array of longitudinal predictor categories, incorporating comprehensive and high-quality data sourced from national registers. Particularly noteworthy is extensive socioeconomic information, which was limited in previous studies. Third, we hypothesized that a score capable of predicting instantaneous mortality risk may offer deeper insights into aging mechanisms compared to longer-term mortality prediction models. Previous studies using omics data encountered challenges because of limited mortality instances for training robust predictors of short-term mortality^6–8^. By using extensive medical, sociodemographic and geographical data collected longitudinally from millions of individuals, we developed a digital aging clock tailored to predict short-term (1-year) mortality. In alignment with the concept of molecular aging clocks, we refer to our approach as a digital aging marker or clock, reflecting the use of secondary electronic data from healthcare and welfare systems, as opposed to molecular markers. However, given our model’s focus on mortality prediction rather than age estimation, the term digital mortality marker may be a more accurate depiction.

Ultimately, our exploration enabled differentiated predictions at a level of detail previously unattainable. For instance, we harnessed detailed economic data to identify disadvantaged individuals, thereby enriching our comprehension of the fairness aspects inherent in aging and mortality clocks.

## Results

### Individuals included in the study, data and model

FinRegistry (https://www.finregistry.fi/) is a comprehensive register-based data resource that provides access to a diverse range of health and sociodemographic data for the entire Finnish population. The unique characteristic of this resource is the breadth of data categories, including healthcare visits, health conditions, medications, surgical procedures, demographic characteristics, welfare benefits, pensions and detailed socioeconomic information (a detailed description of data sources can be found in the Supplementary Information). Notably, some of this information spans decades, dating as far back as the 1970s. The Causes of Death registry is particularly relevant to this study because it offers comprehensive information about death events and causes of death (CODs).

**Fig. 1:**
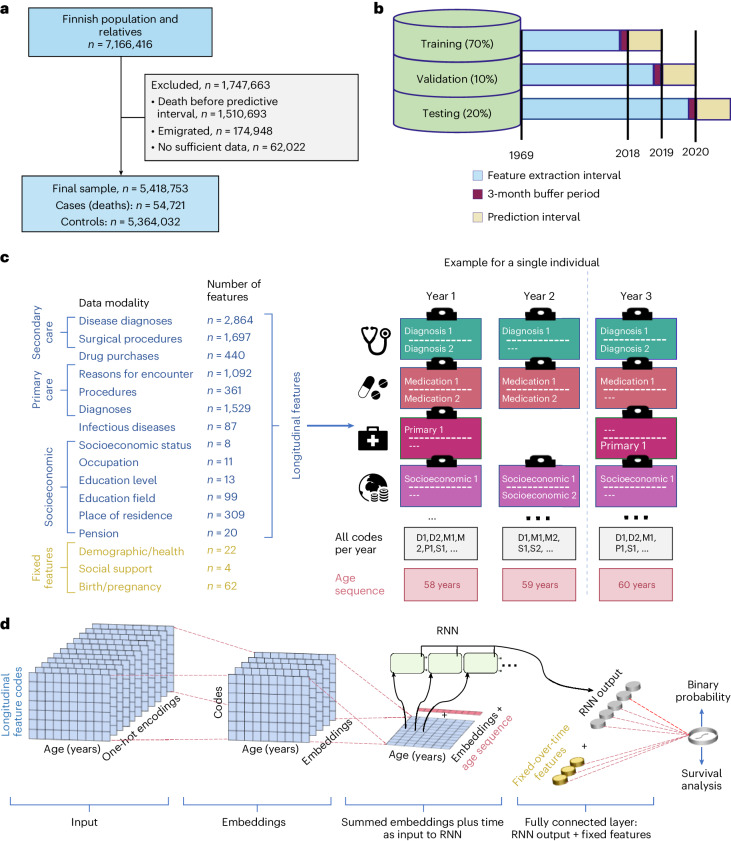
Study population, data and model. **a**, Study population and inclusion and exclusion criteria. **b**, Data division into the training, validation and testing datasets in prospective fashion. **c**, Features included in the model, either treated longitudinally or fixed over time (different types of features and model inputs are color-coded in **c** and **d**) with an example of longitudinal features available for an individual across three years. *n* denotes the number of features in different categories. **d**, Graphical representation of the RNN model. Longitudinal records were embedded and then, together with an age sequence, used as inputs for a recurrent layer. Fixed-over-time features were also added before the output layer.

FinRegistry covers all Finnish residents on 1 January 2010, as well as their parents, spouses, children and siblings. For our study, we included all individuals alive and not emigrated on 1 January 2020 (*n* = 5,364,032; Fig. 1a for a detailed study overview). Our objective was to predict all-cause mortality within 1 year, with approximately 1% of individuals dying within this time frame. To ensure the generalizability of our predictions, we considered three consecutive years for training, validation and testing. Specifically, we predicted mortality in 2018 during training, in 2019 for validation and in 2020 for testing. These shifts ensured that the validation and testing prediction periods remained ‘unseen’ to the model during training (Fig. 1b). The coronavirus disease 2019 (COVID-19) pandemic disrupted the healthcare system in 2020. Therefore, using this year for predictions in our model served as a rigorous ‘stress test’ for assessing its robustness.

To build our models, we used both fixed-over-time and longitudinal features (Fig. 1c). Longitudinal features used coded records exactly as they appeared in the registers while preserving temporal information on the duration between different events. Fixed-over-time features were only used to capture information that was constant throughout an individual’s lifetime, such as basic demographic information. By combining both types of features, we captured both the dynamic and static characteristics of each individual, improving the predictions. Overall, we included 8,620 features, of which 90 were fixed over time and 8,530 were longitudinal.

To capture the complex interactions between events over time, we used a recurrent neural network (RNN) with a gated recurrent unit (Fig. 1d). RNNs are effective in modeling patients’ health histories^28^ and have demonstrated comparable performance to other sequential DL models, such as transformers, in predicting clinical events^29,30^.

To evaluate our DL model against a simpler baseline model, we used a logistic regression model that included only age and sex as predictors of mortality.

### Descriptive results

**Fig. 2:**
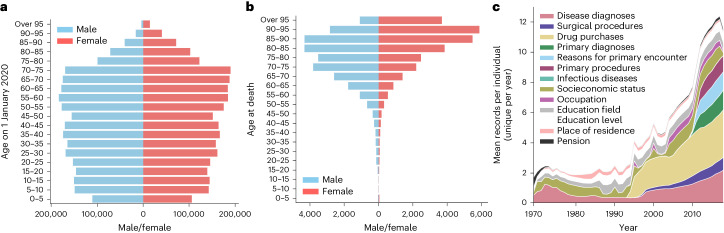
Descriptive results in the testing dataset. **a**, Pyramid plot showing age and sex distribution for a full study population at the start of the predictive interval on 1 January 2020. **b**, Pyramid plot showing age and sex distribution for individuals who died during the predictive interval (the year 2020). **c**, Distribution of the average yearly number of records per individual over time within the testing set. For each individual, duplicate records within a single year were not included. Source data

We explored age and sex distribution in our data as crucial factors influencing mortality (Fig. 2a,b). The mean age of our study population was 44.4 years on 1 January 2020, and there were more females (50.8%) than males (49.2%). The mean age at death, in 2020, was 79.7 years (83.3 for females and 76.1 for males); only 13.2% of deaths occurred before 65 years of age.

**Extended Data Fig. 1:**
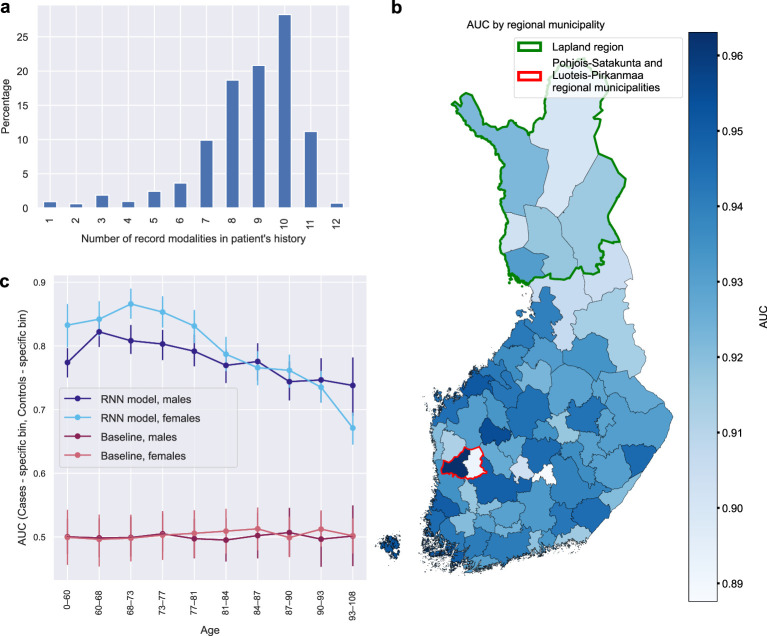
Overview of data records, regional AUC variation, and AUCs for age/sex subgroups. **a**, The number of different types of records (categories) for every individual. Most individuals (78%) had records from 7 to 11 categories. Education field and level were combined into a single category since they were always recorded together. **b**, Variation the AUCs by the regional municipality in Finland. This figure is like Fig. 5a, but the AUC results presented here are from a model that was trained without using geographic (place of residence) features. The purpose of this was to investigate whether observed geographic differences were due to the model’s awareness of geographic information. The green border marks Lapland region in which AUC remained significantly lower than in the rest of Finland, *P* = 0.004, Red border surrounds two neighbouring regional municipalities with still significantly different AUCs, *P* = 0.002. Statistical significance was assessed using permutation testing. **c**, This figure is comparable to (Fig. 4c) and displays areas under the receiver operating characteristic curves (AUC) within specific age/sex subgroups (with cases and controls from a specific subgroup only). However, here in each bin the controls were matched with respect to age (with a precision level of 1 year). Additionally, the number of controls used was the same as the number of cases. The 95% confidence intervals for the AUC were estimated using 1000 bootstrap resamples, determining the 2.5th and 97.5th percentiles of the resulting AUC distribution. RNN = recurrent neural network. The p-value for the difference in AUCs was determined using permutation testing with 1000 iterations, randomly permuting group labels to compute the distribution of AUC differences. Source data

We explored the amount of longitudinal data available over time (Fig. 2c). There was a gradual increase in the mean number of records available per individual over time, with some data categories starting in later years. Specifically, the drug purchase register was introduced in 1995, followed by the outpatient register (reflected in the disease diagnoses and surgical procedures categories) in 1998 and finally the primary care register in 2011. Overall, most individuals had information from multiple feature categories, with 78% of individuals having records for at least eight categories (Extended Data Fig. 1a).

### Model performance

**Fig. 3:**
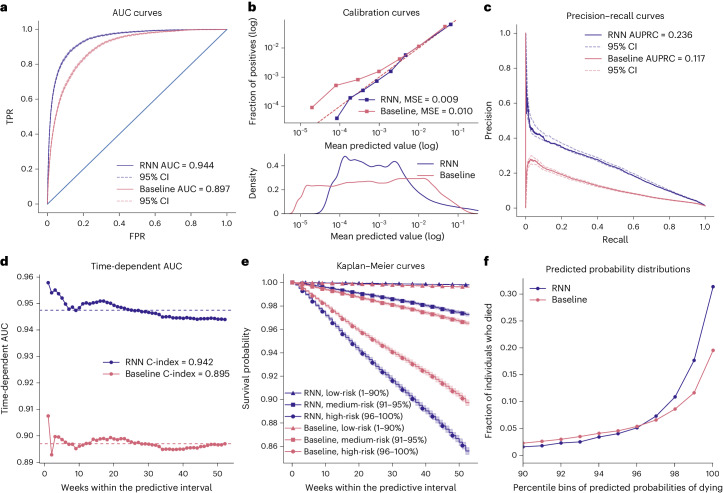
One-year mortality prediction results. **a**, AUC for the RNN and baseline models. **b**, Calibration curves for the RNN and baseline models. Observed and predicted probabilities of death for each risk decile are reported on a log scale because of a skewed probability distribution, with most values close to zero. This is evident in the bottom panel showing predicted probability densities for both models. A quantile binning strategy for calibration curves was used to ensure an equal number of samples in each bin. **c**, Precision–recall curves and AUPRC values for the two models. **d**, Time-dependent AUC curves and C-indexes for each of the 52 weeks in the predictive interval. The dashed lines show the mean AUC for each model. **e**, Kaplan–Meier curves for predicted low-mortality, medium-mortality and high-mortality risk groups in the testing set for two models. Stratification of individuals to the risk groups is according to their predicted survival over time within the predictive interval. Although the low-risk group covers a large 1–90 percentile range, the curves are nearly horizontal and overlap, with low mortality over time for both models. The shaded areas show the exponential Greenwood confidence interval. **f**, Fractions of individuals who died in the testing set as a function of percentile bins of predicted mortality probabilities within the predictive interval for the two models. We have only plotted individuals at medium and high risk (90+ percentile). In **a** and **c**, the 95% CIs were estimated using 1,000 bootstrap resamples, determining the 2.5th and 97.5th percentiles of the resulting AUC distribution. Source data

The RNN model included 2.9 million trainable parameters and achieved an area under the receiver operating characteristic curve (AUC) of 0.944 (95% confidence interval (CI) = 0.942–0.946) for binary classification, surpassing the baseline model that relied solely on age and sex, which achieved an AUC of 0.897 (95% CI = 0.894–0.899; Fig. 3a). Additionally, the RNN model exhibited superior calibration, as indicated by a lower mean squared error (MSE) between predicted values and true labels (Fig. 3b). The RNN model achieved a higher area under the precision–recall curve (AUPRC) than the baseline model (0.223 versus 0.119; Fig. 3c). It is worth noting that the AUPRC is influenced by the degree of class imbalance and is expected to be lower in situations where class imbalance is high, as observed in our study.

When we considered time-to-death rather than binary classification, the RNN yielded a C-index of 0.942 (95% CI = 0.940–0.944). While the RNN model demonstrated slightly better performance in predicting mortality at the start of the year, it maintained a consistently high C-index throughout the entire predictive interval (Fig. 3d).

We compared the Kaplan–Meier curves for three risk groups categorized according to the predicted mortality probability from either the RNN or the baseline model (Fig. 3e). The RNN model showed a larger disparity in survival rates among the three groups, compared to the baseline model. For instance, the high-risk group, consisting of individuals with predicted mortality probabilities ranging from the 96th to 100th percentile (that is, 5% of the individuals with the highest predicted risk), exhibited a mortality rate of 16.8% by week 52, compared to 11.4% predicted by the baseline model (Fig. 3f). To put it differently, the RNN model predicted 69.5% of all deaths that occurred in the testing dataset to be in the high-risk group, compared to the baseline model’s prediction of 49.6% of all deaths. Overall, the RNN model outperformed the baseline model in differentiating between medium-risk and high-risk groups.

We compared the performances of the RNN model with penalized logistic regression and XGBoost^31^ models trained with the same 8,530 longitudinal features but expressed as binary variables indicating the presence or absence of a record in individuals’ registry history. After parameter optimization, we observed AUCs of 0.934 and 0.938 for logistic regression and XGBoost, respectively, which was lower than the RNN model (AUC = 0.944).

### Model performance according to COD and age

To test the robustness of the model across different medically and socioeconomically relevant groups, we first examined groups based on different COD and age. We took two different approaches.

The first approach is group identification, which evaluates the predictability or identifiability of a specific subgroup within the entire population. Previous studies used this approach to compare the predictability of different diseases^32^, or the subtypes of diseases^33,34^, within the pool of healthy individuals.

The second approach is group differentiation, which compares prediction performance within a particular subgroup of the population relative to another subgroup from the same population (for example, a specific age group). This approach is typically used in algorithm fairness studies to assess differences in prediction performance between groups defined by ethnicities, sexes, ages and other attributes. Researchers in aging also use this approach to evaluate the efficacy of biological age predictors beyond what is solely accounted for by chronological age in different age groups^25,35^.

**Fig. 4:**
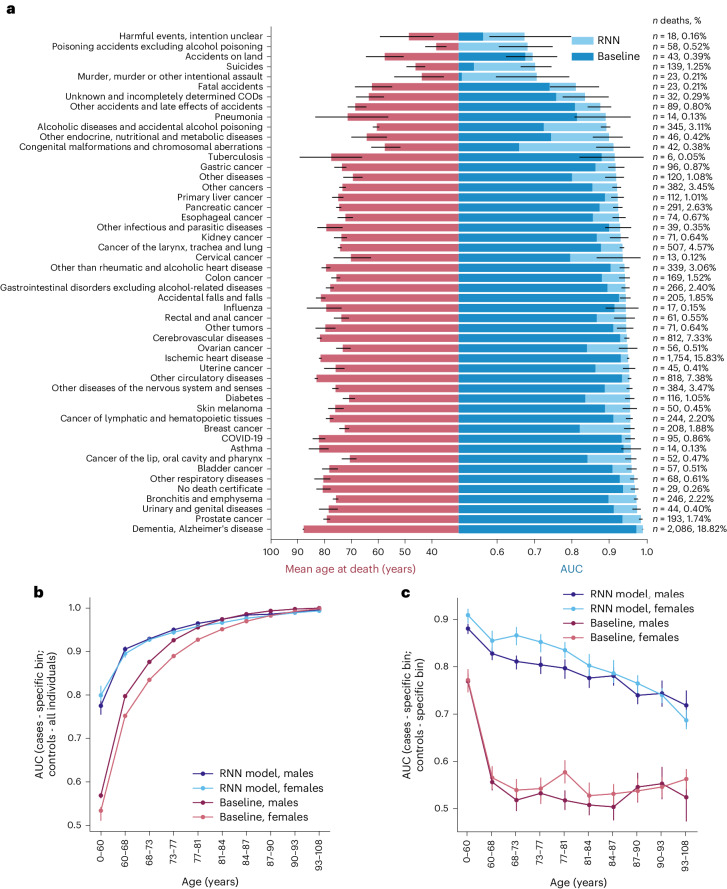
Mortality prediction for the cause-of-death, age and sex subgroups. **a**, Left, Average age at time of death from a specific cause within a testing set. Right, AUC values for individuals dying from a specific cause. AUCs are reported separately for the RNN and baseline models. CIs obtained via bootstrapping are reported only for the RNN model to improve readability. The number and percentage of people dying from a specific cause is given in the right margin of a panel. Only CODs with five or more cases were considered. **b**, Model AUC scores for specific age and sex subgroups of cases (group identification approach: considering cases from a specific age and sex subgroup versus all controls). **c**, Model AUC within specific age and sex subgroups (group differentiation approach: considering cases and controls from a specific subgroup only). This corresponds to evaluating the predictive performances of the model after the effect of age as a predictor has been substantially minimized (for more precise removal of the age effect, see Extended Data Fig. 1c). Within the ten age bins in **b**,**c**, an equal number of cases was ensured. The 95% CIs were estimated using 1,000 bootstrap resamples, determining the 2.5th and 97.5th percentiles of the resulting AUC distribution. Source data

We used the group identification approach to compare mortality prediction across 50 different CODs (five CODs were excluded because of an insufficient number of cases of five or fewer; Fig. 4a). The frequency of different CODs varied substantially, ranging from less than 1% for external CODs (such as accidents or suicides) to 15.8% and 18.8% for the most common CODs, namely ischemic heart disease and dementia, respectively (rightmost part of Fig. 4a). The RNN model showed good predictive performance across CODs, achieving an AUC of over 0.8 for 45 of 50 CODs. The prediction performances for CODs related to accidents and violence were substantially lower than disease-related CODs (average AUCs of 0.761 and 0.939, respectively). Nonetheless, the RNN model substantially outperformed the baseline model, especially for CODs related to accidents and violence, with a mean AUC improvement of 0.11 (light blue bars in Fig. 4a). It is worth noting that COVID-19 emerged as a new COD in 2020; although the RNN model was not designed to predict COVID-19 mortality because of the absence of COVID-19 deaths in the training data, it achieved a high AUC of 0.956.

Both the RNN and baseline models demonstrated better predictions for CODs occurring at older ages. For instance, individuals who died from dementia at a mean age of 87.9 years were well predicted by both models (AUC = 0.989 and 0.971 for RNN and baseline, respectively). Conversely, the RNN model was substantially better at predicting CODs occurring among younger individuals. For example, suicide (mean age = 46.3) was substantially better predicted by the RNN compared to the baseline model (AUC = 0.702 versus AUC = 0.539). Overall, the mean age at death was the primary factor contributing to differences in AUC for the baseline model (*R*^2^ = 0.992), whereas this association was weaker for the RNN model (*R*^2^ = 0.809). Interestingly, there was no discernible relationship between the prevalence of each COD and prediction performance, with both rare and common CODs achieving high AUCs (*R*^2^ = 0.091 and 0.057 for the baseline and RNN models, respectively).

As COD predictability showed a strong correlation with age, we further explicitly explored the relationship between model performance and age at death. We used both the group identification (Fig. 4b) and group differentiation (Fig. 4c) approaches to explore this relationship in detail and compare the approaches.

First, using the group identification approach, we explored how well a model identified individuals who died within a specific age bin among the entire population, irrespective of their age. The results mirrored those of the COD analyses, with both RNN and baseline models exhibiting better predictions for the older age groups. The RNN model performed notably better, particularly in the bins of the youngest individuals (Fig. 4b).

Second, we used a group differentiation approach and assessed model performance limiting cases and controls to a specific age bin (Fig. 4c). This corresponds to evaluating the predictive performances of the model after the effect of age as a predictor has been substantially minimized. In contrast to the group identification task, the RNN model’s prediction performance declined in the older age bins, showing higher performance for young females than young males. For the baseline model, performance was at a random guessing level (AUC ~0.50) in each age bin, except for the youngest age group with the widest age range and not sufficient control for age differences between cases and controls. After exactly matching the age and sex of cases and controls within each age group, the baseline model, but not the RNN model, showed random guessing level performance across all age groups (Extended Data Fig. 1c).

### Prediction fairness

We examined the fairness of predictions by comparing model performance across groups of individuals based on geographical location, monthly pension level and other sociodemographic variables.

**Fig. 5:**
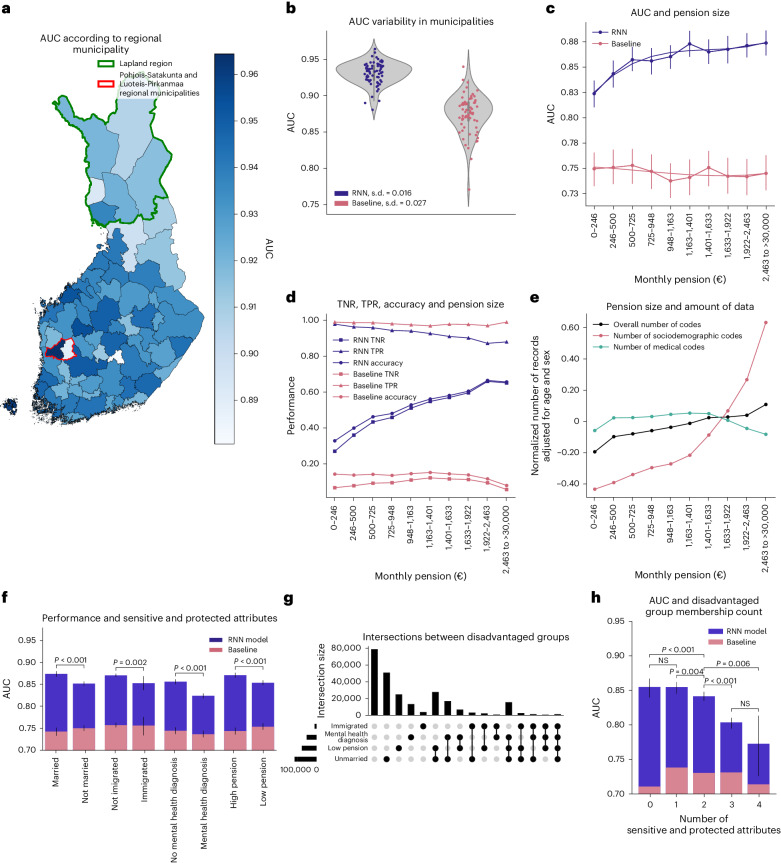
Fairness regarding place of residence, pension size and other sensitive attributes. **a**, AUC variation according to regional municipality in Finland. The green border marks the Lapland region in which the AUC was significantly lower than in the rest of Finland, while the red border surrounds two neighboring regional municipalities with significantly different AUCs. **b**, Each dot represents a different regional municipality. Variability in prediction performance in different regional municipalities showed a larger spread and greater geographical variability for baseline compared to the RNN model. **c**, AUC from the baseline and RNN models within each pension level bin. The RNN model had higher prediction performances among individuals with a higher pension. **d**, Accuracy, TPR and TNR for the RNN and baseline models as a function of pension. The classification metrics were calculated based on a probability cutoff of 0.0089 for the RNN model and 0.0094 for the baseline model (see Methods for the cutoff calculation). For an RNN model, an increase in AUC with greater pension size was driven by TNR—better identification of individuals who did not die during a predictive interval. **e**, The average number of total records available for training the RNN model as a function of pension size. The average number of total records per individual was adjusted for age and sex and then normalized. This metric allows the evaluation of whether individuals with a higher pension have more information available, potentially explaining the better performance of the RNN models. Records from three main data categories are reported. In **c**–**e**, ten pension bins were used, ensuring an equal number of cases in each. **f**, AUCs for different attributes considered protected or sensitive: marital status, immigration status, mental health diagnosis and pension size (individuals were split into two pension size groups, thus assuring an equal number of cases in each). **g**, UpSet plot^36^ visualizing the intersections between four groups of disadvantaged individuals. **h**, AUC for the RNN and baseline models in individuals having none, one or several disadvantages across four sensitive and protected attributes simultaneously. The 95% CIs were estimated using 1,000 bootstrap resamples, determining the 2.5th and 97.5th percentiles of the resulting AUC distribution. The *P* value for the difference in AUCs was determined using permutation testing. Source data

First, we compared the RNN model performance across different regional municipalities. We found notable variability in prediction performance between different regional municipalities, with AUCs ranging from 0.881 to 0.964 (Fig. 5a). For example, we observed lower prediction performance in the northern Lapland region, consisting of six regional municipalities, compared to the rest of Finland (AUC = 0.924 versus 0.939, *P* = 0.002). Substantial differences were observed between neighboring regional municipalities. For example, Pohjois-Satakunta and Luoteis-Pirkanmaa, despite their geographical closeness, had significantly different model performances (AUC = 0.964 versus 0.890, *P* < 0.001). The differences were partly explained by population density as we observed a positive correlation (*r* = 0.23, *P* = 0.05) between population density and AUC in different regional municipalities. To determine whether the observed variability in AUC was influenced by the model’s awareness of geographical information, we retrained the RNN model without geographical features, but we still observed similar differences in performance (Extended Data Fig. 1b). The baseline model had higher variability in its prediction performance across different regional municipalities compared to the RNN model (s.d. in AUC of 0.027 versus 0.016; Fig. 5b).

**Extended Data Fig. 2:**
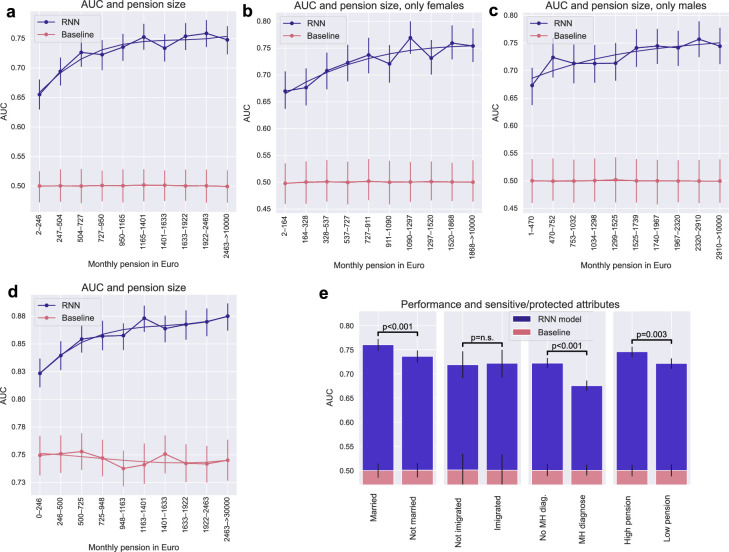
AUC analysis of age/sex matching for pension and protected attributes. **a**, This figure is comparable to Fig. 5c but here controls were matched for age (with a precision level of 1 year) and sex within each bin. Additionally, the number of controls used was the same as the number of cases. Separate curves were plotted for female (**b**) and male (**c**) samples. Despite the substantial disparity in pension distribution between sexes, both sexes exhibited comparable AUC differences between different pension bins using the recurrent neural network (RNN) model, as observed in Fig. 5c. In contrast, the performance of the baseline model approximated random guessing ( ≈ 0.5). **d**, This figure is like Fig. 5c, but the AUC results presented here are from a model that was trained without using pension features. The purpose of this was to investigate whether observed differences in different pension bins were due to the model’s awareness of pension information. AUCs differences between pension size subgroups remained similar as in Fig. 5c. **e**, This figure is comparable to Fig. 5f, showing AUCs for different attributes considered protected or sensitive, namely marital status, immigration status, mental health (MH) diagnosis, and pension size (individuals were split into two pension size groups assuring an equal number of cases in each). However, in this analysis, socially disadvantaged and advantaged groups were matched for a number of samples, age (with a precision level of 1 year), and sex. Additionally, the number of samples, age, and sex of controls were matched to the corresponding cases. AUC difference by immigration status became non-significant, likely due to the matching process. This outcome can be attributed to the low number of immigrated individuals who were cases, resulting in limited statistical power. The p-value for the difference in AUCs was determined using permutation testing with 1000 iterations, randomly permuting group labels to compute the distribution of AUC differences. The 95% confidence intervals for the AUC were estimated using 1000 bootstrap resamples, determining the 2.5th and 97.5th percentiles of the resulting AUC distribution Source data

Second, we investigated the fairness of our mortality prediction model with respect to average monthly pension levels in 2020. We chose old-age pension because it is based on an individual’s income throughout their working life and is particularly relevant among older individuals, where most deaths occur. To focus our analysis, we limited our investigation to individuals over 65 years of age because this group accounted for 85% of all deaths in 2020, and 93% in this group received an old-age pension. There was a clear positive relationship between pension levels and AUC for the RNN model, with a higher AUC for higher pension (for example, AUC = 0.824 for a pension between 0 and 246€ per month versus AUC = 0.874 for a pension between 2,463 and more than 30,000€ per month, *P* < 0.001). No such relationship was observed for the baseline model (Fig. 5c). Similar results were also observed after matching individuals for age and sex within each pension bin (Extended Data Fig. 2a–c) and when a model was retrained without pension features (Extended Data Fig. 2d). Analysis of sensitivity (true positive rate (TPR)) and specificity (true negative rate (TNR)) revealed that the increase in AUC with greater pension size was predominately driven by increasing TNR (that is, better identification of individuals who did not die during the predictive interval; Fig. 5d). We also explored whether differences in the amount of training data could have influenced AUCs in different pension bins (Fig. 5e). Individuals in higher pension bins tended to have more socioeconomic records, while the number of medical records and an overall number of records was similar within different pension bins.

Third, we expanded our fairness analyses to four sensitive and protected attributes, which partially overlapped (Fig. 5g). We found that AUCs for sensitive and protected groups, such as those who were unmarried, had immigrated, had mental health diagnoses or received low pensions, were significantly lower than for their counterparts (Fig. 5f; *P* < 0.002 for all comparisons). We also performed the same comparisons after matching for age and sex within socially disadvantaged and advantaged groups: the observed effects remained, except for immigration status (Extended Data Fig. 2e). Additionally, belonging to multiple sensitive and protected groups simultaneously resulted in considerably worse AUCs (Fig. 5f) in the RNN, but not in the baseline model. Refer to the UpSet plot^36^ for the sample sizes and intersections of the four disadvantaged groups (Fig. 5h).

### Model explainability

**Fig. 6:**
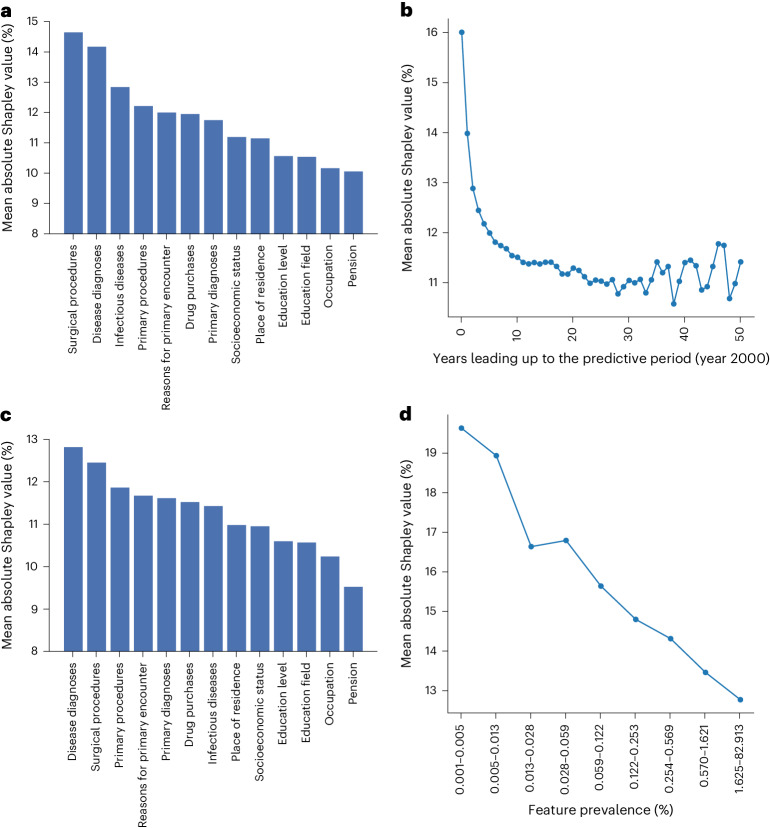
Feature importance using Shapley values. **a**, Mean absolute Shapley values within each feature category. **b**, Mean absolute Shapley values within each year leading up to the predictive period (year 2020). **c**, Mean absolute Shapley values within each feature category excluding all feature codes reported in the last 5 years before the predictive period. **d**, Mean Shapley values across features classified within different prevalence bins. For bar-and-whisker plots, see Extended Data Fig. 4. Source data

**Extended Data Fig. 3:**
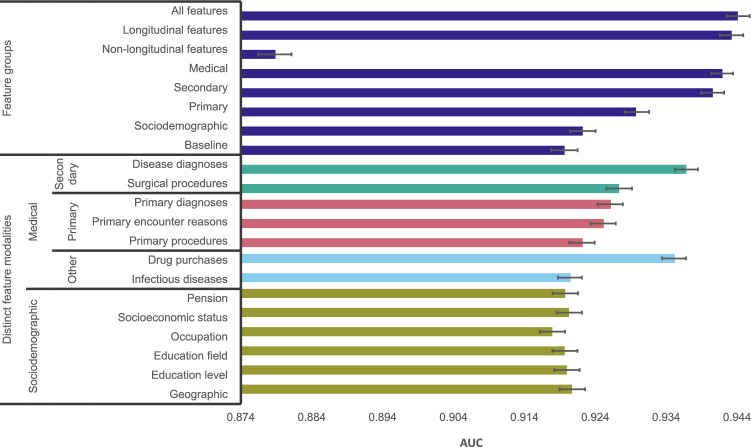
Importance of distinct feature categories and groups of categories. Importance was measured by randomly permutating all features except the ones contained within a distinct category or group of categories. Then the recurrent neural network model was used for classification and areas under the receiver operating characteristic curves were compared. Within feature groups, longitudinal features comprised medical and socioeconomic features. In turn medical features comprised secondary and primary care features. Non-longitudinal features included fixed over time features. The baseline performance is obtained by permuting all features, which limits the information provided to a model to a count of features per age year, while specific feature information for all features is permuted. Classification performance for each individual category is in the lower part of the figure. The 95% confidence intervals for the AUC were estimated using 1000 bootstrap resamples, determining the 2.5th and 97.5th percentiles of the resulting AUC distribution Source data

We used Shapley values^37^ to evaluate the contribution of each of the 8,530 longitudinal features, both individually and aggregated within data categories, expressed as a mean absolute change in predicted mortality probability from an individual-specific Shapley baseline where no longitudinal features were included. We found that feature categories related to surgical procedures and diagnoses recorded in secondary care had the highest Shapley values (Fig. 6a). In contrast, socioeconomic features demonstrated a lesser impact. We used an alternative test, permutating all features except those within a specific category, to identify which categories made the largest contribution to mortality prediction. The findings largely aligned with the Shapley results, indicating that secondary care features held the highest importance, followed by primary care features, with socioeconomic features ranking the lowest (Extended Data Fig. 3). We also observed that features measured closer to the predictive period held a more pronounced influence on predictions than features measured earlier in an individual’s registry history (Fig. 6b). To delve deeper into this aspect, we conducted additional analysis by excluding the last 5 years leading up to the predictive period. The objective was to investigate whether the same feature categories were important during the earlier stages of an individual’s registry history. Contrary to our hypothesis of a larger role of socioeconomic features, the results demonstrated a similar trend as in the main analysis, with secondary care surgical procedures and diagnoses showing the largest Shapley values (Fig. 6c). Finally, we considered the impact of longitudinal features according to how commonly they were observed in the study population. We found that more rare features had higher Shapley values (Fig. 6d) probably because these features tend to reflect more distinct alterations in a patient’s medical and socioeconomic trajectory. Focusing on the top 100 features with the highest impact at a population level (prevalence of at least 0.1%), 48 were linked to secondary disease diagnoses, particularly those related to substance abuse and impacting the central nervous system; 39 were associated with secondary surgical procedures, where several cancer treatments predominated; nine pertained to drug purchases; and the remaining four were associated with primary healthcare codes (Supplementary Information and Supplementary Table 4).

## Discussion

In this study, we used a nationwide, high-quality, multi-category dataset to predict 1-year all-cause mortality for the entire Finnish population and to investigate variability in predictions and fairness at a level of detail not previously possible. Our prediction score can be interpreted as a digital aging clock specifically designed to predict short-term (1-year) mortality.

The model exhibited strong predictive abilities (AUC = 0.944, 95% CI = 0.942–0.946) and was well calibrated, surpassing a simpler baseline model. For example, a substantial proportion of all deaths (69.5%) occurred in a high-risk group including only 5% of individuals with the highest predicted risk. Strong performance was observed despite using a prospective testing approach to ensure that the prediction period remained ‘unseen’ by the model during training. The model could be flexibly applied across different ages and cause-of-death groups, including previously unseen CODs such as COVID-19. Notably, our model demonstrated a substantial improvement over the baseline model when predicting deaths resulting from accidents or violence. We speculate that the inclusion of socioeconomic features may have aided in predicting such seemingly external CODs.

**Extended Data Fig. 5:**
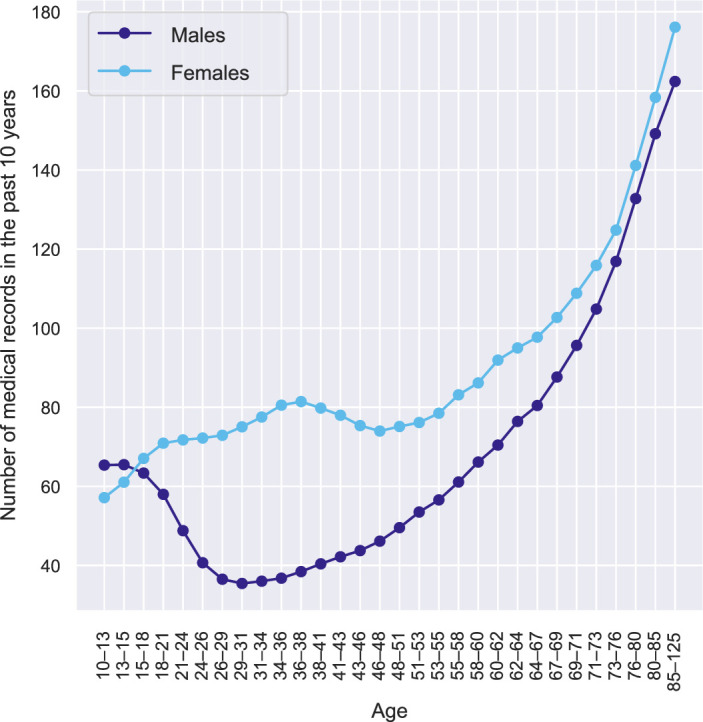
Amount of data available for different sexes. Number of medical records recorded from 2010 to 2019 (last 10 years in individuals’ medical history), divided by gender, in a test sample. Source data

Even after removing the effect of chronological age, which is the strongest mortality predictor, our model achieved an AUC of 0.769 for males and 0.822 for females aged 0–60 (Extended Data Fig. 1c). This additional predictive performance beyond chronological age suggests the potential of our model as a digital marker of biological age. In comparison, markers of biological age, such as frailty indexes, DNA methylation and telomere length, achieve lower performance for mortality prediction^38–40^. Intriguingly, our model exhibited stronger predictive performance among younger, but not older females, compared to males. As we observed greater contact with healthcare among younger females, partially because of childbirth, compared to males (Extended Data Fig. 5), we speculate that this may provide predictive information that is not available for males.

After controlling for chronological age, our model’s performance gradually decreased in older individuals. As people age, they start to differ more from each other because they experience biological and environmental changes at varying rates and degrees^1^. This increases variability in functional abilities, such as mobility, self-care, ability to perform usual activities, pain and discomfort, and anxiety and depression^41^. Furthermore, the combination of increased damage and reduced resilience can lower the threshold for adverse events to result in mortality^13^. The presence of substantial heterogeneity among older individuals probably diminishes the distinctiveness of data available for individuals who will die in the short term compared to those who will not, thereby complicating the accuracy of predictions.

The biomedical and human genetics field has studied model fairness extensively^42–44^, but most studies lack information on sensitive and protected attributes. While electronic health records provide ample information on ethnicity and ethnic grouping, other socioeconomic characteristics are often unavailable. This limitation leads to a focus on fairness considerations based primarily on ethnicity, ethnic grouping, age and sex in most studies. Our study breaks new ground by comprehensively evaluating fairness across multiple, including multilevel, sensitive and protected attributes. We selected several attributes that are highly valued in Nordic European societies and are applicable more broadly, including geographical equality, income, marital status, immigration status equality and destigmatization of mental health diagnoses. For all these attributes, we found significantly worse model performance for disadvantaged groups using the RNN model, while none of the differences were significant for the baseline model. Moreover, we observed that being disadvantaged in multiple ways at the same time resulted in substantially worse prediction performance. Several factors, including those considered as sensitive and protected attributes, are not equally distributed between less densely populated regions compared to more populated regions. For example, previous research suggested that healthcare quality is lower in less densely populated regions^45^, indicating a potential influence on regional disparities. In our study, we observed a positive yet weak association (*r* = 0.23, *P* = 0.05) between population density and AUCs in different regional municipalities.

Different hypotheses have been proposed to explain why prediction models perform worse for disadvantaged groups across sensitive and protected attributes. One possible explanation is that there are fewer cases in the disadvantaged group, leading to less power during model training^46^. Another explanation is that disadvantaged individuals have lower contact and poorer quality of healthcare, resulting in missing data and measurement errors, ultimately skewing the predictions^20^. Differences in age and sex between socially advantaged and disadvantaged groups could also be an underlying driver of the observed differences in prediction performance, as well as the explicit inclusion of the sensitive and protected attribute as a feature in the model^18^. We thoroughly investigated all these hypotheses by analyzing the differences in AUC among monthly pension levels, yet we could not identify the culprit of the variation. We ensured that the number of cases was equal in each bin; the inclusion of pension information in the model, as well as differences in age and sex distribution between pension bins, did not change the results. While we observed a higher number of socioeconomic records for individuals at higher pension levels, the number of medical records, which contribute more to predictive performance, remained comparable across different pension size bins. One possibility is that receiving a higher pension is associated with reduced heterogeneity and entropy. This means that individuals who receive a higher pension may be more similar to each other in terms of the contribution of different features to mortality prediction. This also means that cases (that is, individuals who died within the next year) may stand out more because of reduced heterogeneity among controls. This could allow the model to better differentiate between cases and controls, resulting in more accurate predictions.

Our study has several limitations. First, we did not validate the model outside Finland, highlighting the need for replication in other countries. It would be particularly valuable to assess prediction fairness for socioeconomically disadvantaged groups in different countries, given that Finland has relatively low poverty rates and socioeconomic inequality, as evidenced by a low Gini index^47^. Second, our model lacks biological or genetic markers, self-reported lifestyle information and other data commonly available in epidemiological studies, but not collected nationwide. The integration of these markers could further improve model performance. Third, we did not consider other DL model architectures beyond RNNs; however, we used simpler models, that is, penalized logistic regression and XGBoost. Previous work showed that RNNs have comparable performance to other sequential DL models in predicting clinical events^29,30^. More work is needed to identify models for aging clocks that can balance interpretability, fairness, scalability and prediction performance. Fourth, most of the fairness analyses were limited to individuals aged 65 and older and to a limited number of sensitive and protected attributes. It is currently unclear what the optimal set of sensitive and protected attributes should be, particularly given the considerable overlap observed in our population. A multidisciplinary approach that includes social scientists and legal experts may be necessary to identify widely available attributes for which artificial intelligence (AI)-based model fairness should be assessed.

In conclusion, our study demonstrates how DL can effectively leverage longitudinal multi-category nationwide information to accurately predict short-term mortality risk and derive a digital aging clock. The model performed well across different CODs. Future studies should evaluate how probability scores obtained from this model relate to overall health, clinically relevant features and outcomes, as done in recent work on a digital marker of coronary artery disease^48^. While there is clear potential for such models, it is important to assess their performance among population groups that already carry the greatest disease burden. We have presented an in-depth examination of fairness at a national scale and revealed that model performance was significantly lower among disadvantaged individuals across multiple sensitive and protected attributes. Therefore, we recommend that studies developing and testing AI models in biomedicine should consider algorithm fairness, entertaining greater integration between socioeconomic and health data.

## Methods

FinRegistry is a collaboration project between the Finnish Institute for Health and Welfare and the Data Science Genetic Epidemiology research group at the Institute for Molecular Medicine Finland, University of Helsinki. The FinRegistry project has received the following approvals for data access from the National Institute of Health and Welfare (THL/1776/6.02.00/2019 and subsequent amendments), Digital and Population Data Services Agency (VRK/5722/2019-2), Finnish Center for Pension (ETK/SUTI 22003) and Statistics Finland (TK-53-1451-19). The FinRegistry project has received institutional review board approval from the National Institute of Health and Welfare (Kokous 7/2019).

### Study population

The FinRegistry dataset includes 7,166,416 individuals of whom 5,339,804 (74.51%) are index individuals (every resident in Finland alive on the 1 January 2010) and the remaining 1,826,612 are relatives (offspring, parents, siblings) and spouses of the index individuals, who are not index individuals themselves.

### Inclusion and exclusion criteria

The final sample of this study included alive and not emigrated individuals (*n* = 5,418,753; Fig. 1a). From an initial sample of 7,166,416, we excluded 1,510,693 individuals who died before the predictive intervals of the training, validation and testing datasets (Fig. 1b), 174,948 individuals who emigrated and 62,022 individuals who never interacted with healthcare, purchased drugs or had any entries in socioeconomic registers. These individuals were probably living abroad; given the underreporting of emigration events (especially within Europe), we excluded these individuals from the study.

### Outcome definition

Our main outcome of interest was mortality. The FinRegistry project has information about individuals’ deaths from two registers: Statistics Finland COD and the relatives register from the Digital and Population Data Services Agency. For our purposes, we considered individuals as deceased if either the year of death was recorded in the Statistics Finland death register (the year was used because for a small proportion of entries only year but no exact date was available) or the date of death was recorded in the Digital and Population Data Services Agency relatives register. Both registers do not fully overlap, with larger disagreement in earlier years and considerably smaller in later years. For the period after 1 January 2018, there was a good agreement between the two registers (99.83%).

As cases, we considered 54,721 individuals who died during the predictive intervals of the training, validation and testing sets (Fig. 1b). The remaining 5,364,032 were alive during those periods and were considered controls, with a 1.02 case per 100 controls.

### Definition of the training, validation and testing datasets

We randomly split the study population into three groups, training (70%), validation (10%) and testing (20%; Fig. 1b). The first records in the registers used in this study started on the 1 January 1969 (the start of the cancer register). Thus, for training purposes, the predictors were considered from the 1 January 1969 until a predictive interval that was different for each of the data splits. The validation and testing intervals were shifted 1 year forward each to allow some external validation in terms of time, leaving validation and testing prediction periods ‘unseen’ to a model during training. This resulted in feature extraction intervals lasting until 30 September 2017 for training, 30 September 2018 for validation and 30 September 2019 for testing. To increase model generalizability, we used an external temporal validation approach, where the predictive intervals used to define cases and controls were different for training (1 January 2018 to 31 December 2018), validation (1 January 2019 to 31 December 2019) and testing (1 January 2019 to 31 December 2019). Before each predictive interval, we also left a 3-month buffer period (where data were not used for training), to avoid potential outcome information leakage into the training data.

### Features

Both longitudinal and fixed-over-time features were considered, with a preference for a longitudinal format that retains more information. Longitudinal features included medical, sociodemographic and geographical records, while fixed-over-time features included various information predominantly about demographics and health (Fig. 1c). For a detailed description of these features, see the Supplementary Information.

### Data preparation and missing data treatment

We kept our data curation to a minimum, largely using all medical and sociodemographic records as they appear in the original registers to facilitate transferability and avoid biases that may be introduced with feature engineering. For fixed-over-time features, missing values in continuous and ordinal variables were replaced with mean and mode; an additional binary variable denoting missingness was created. For categorical variables, a category denoting missingness was created. All features were standardized.

### Longitudinal features

For every individual, we considered age as a timescale. That is, all records observed within each year of age were grouped together. The right side of Fig. 1c shows an illustrative example of how medical and sociodemographic records from each year of an individual’s register history were collated to form sequences used as model inputs. Only unique records within each age year were retained to form a vector of length 100. For a small portion of age year bins (0.03%) that exceeded 100 unique records, a random subsample of 100 values was used; zero padding was used for years with fewer than 100 records.

### Fixed-over-time features

Fixed-over-time features consisted of categorical, continuous and ordinal features that did not change over time and were not used in a longitudinal fashion within the model. They were instead added separately before the last layer of the model (Fig. 1d).

### Models

A good model for sequential health and socioeconomic data should be able to capture complex interactions between records over time. Where the amount of data, sparsity and time windows between records can substantially differ between individuals and records could be repeated multiple times. These complexities resemble the challenges also faced in natural language processing as individual life events resemble individual words in natural language. Thus, we used an RNN, namely a gated recurrent unit, which performed similarly or better than a transformer and other commonly used models with sequential DL architecture for clinical event predictions^28,29,49^. Longitudinally expressed records after embedding individuals’ lives year by year were used as inputs to a recurrent layer (Fig. 1d). DL analyses were implemented with PyTorch^50^. We also trained penalized logistic regression and XGBoost^31^ models. We used the same 8,530 longitudinal features as with the RNN but expressed them as binary variables denoting either that a record existed or did not exist in individuals’ registry history. We followed the TRIPOD recommendations for prediction model development and reporting (Supplementary Table 5).

### Hyperparameter optimization

For RNN hyperparameter tuning, we used the Tree-structured Parzen Estimator algorithm implemented within the hyperparameter optimization framework Optuna^51^. For the RNN models, we optimized six parameters with the objective of maximizing the AUC in the validation dataset. In all the reported analyses, we used the models with an optimized learning rate of 0.0004, weight decay (L2 penalty) of 7.4 × 10^–6^ and a dropout rate of 0.46 used in a dropout layer following the RNN layer. The embedding dimension and hidden layer size were 250 and 250, respectively. For all models, we used a batch size of 200 because it ensured efficient model running given the limited computational resources.

For the penalized logistic regression and XGBoost models, we used grid search and threefold cross-validation to optimize the hyperparameters with an objective to maximize the AUC. For logistic regression, the grid consisted of three parameters: penalty, either L1 or L2, regularization strength *C* in the range (0.001, 0.01, 0.1, 1, 10, 100) and solver liblinear or saga. The selected best parameters were: L1, *C* = 0.1, liblinear. For XGBoost, five parameters were optimized: the learning rate in the range (0.01, 0.05, 0.1), the maximum depth of a tree (3, 5, 7), the fraction of the samples used for fitting the individual trees (0.5, 0.75, 1.0), the fraction of features used for fitting (0.5, 0.75, 1.0) and the number of boosting rounds or trees to build (50, 100, 150), with the selected best parameters being 0.1, 7, 0.75, 0.5 and 150, respectively.

### Baseline model

To evaluate the impact of our DL model on performance when compared to only using age and sex information, we used a logistic regression model without any regularization, using only age and sex as features.

### Calibration curves

To assess the calibration of predicted mortality probabilities, we used calibration curves and compared the mean predicted probabilities of mortality with observed mortality rates within different predicted probability bins. Ten bins were defined, each having an equal number of cases.

### Evaluation of algorithm performance

For the binary prediction evaluation, our main metric was the AUC. This was based on previous literature and clinical recommendations^22,28^. In addition, the AUC is not biased toward any class, meaning that both majority and minority classes are equally important when calculating the AUC. This makes the AUC an attractive choice with imbalanced data. However, it is important to note that AUC can be unreliable when the minority class has an insufficient number of samples. This is because even a small change in the number of correct or incorrect predictions within the minority class can lead to substantial changes in the AUC and AUC score. To address this issue, we only included subsamples that had at least five samples in the minority class in our analyses. The 95% CIs for the AUC error bars were calculated using bootstrapping, a method that estimates the sampling distribution by resampling with replacement from the original data. We performed 1,000 bootstrap resamples and calculated the AUC for each sample. The CI was determined by identifying the 2.5th and 97.5th percentiles of the bootstrapped AUC distribution^52^. To determine the statistical significance of the difference in AUCs between two groups, we used a permutation test with 1,000 iterations. The true AUCs were first calculated for each group. Group labels were then randomly permuted and the AUC difference was recalculated for each permutation. The *P* value was computed as the proportion of permuted AUC differences greater than the observed difference, using a significance level of *P* = 0.05. This approach, based on nonparametric statistics, offers a robust means of hypothesis testing without assuming a specific distribution of the data^53^. For the survival analyses, we report the C-index and time-dependent AUC at any time between the first and 52nd week within a predictive interval. We also split our testing dataset into three risk groups based on predicted mortality probability: low-risk (1–90 percentile), medium-risk (91–95 percentile) and high-risk (96–100 percentile) and compared the survivability of these groups by plotting Kaplan–Meier curves.

### Fairness evaluation

We chose the AUC as our fairness evaluation metric; however, there are many measures that can be used to evaluate fairness, with the equalized odds ratio (OR) being among the most commonly used^18^. While the equalized OR aims to ensure an equal TPR and false positive rate (FPR) between subgroups at a specific probability threshold, AUC parity ensures equal AUCs between subgroups; because the AUC curve is a function of FPR and TPR, the AUC could be seen as the equalized OR at all probability thresholds. Using the AUC is especially beneficial for imbalanced samples, where choosing a specific probability threshold may be arbitrary. To evaluate fairness, the samples were stratified into subgroups based on their protected attributes. For continuous attributes such as age and pension, we divided subsamples into bins ensuring an equal number of cases (individuals who died during a predictive interval) in each subgroup.

The AUC was calculated for each of the stratified subgroups. Additionally, for the pension attribute, we reported accuracy, TPR and TNR. To calculate these measures we used a probability threshold that maximized the geometric mean of sensitivity and specificity: $\max \sqrt{\mathrm{{TPR}}\times (1-{\mathrm{FPR}})}$.

### Model explainability

We assessed the importance of specific features using an RNN model trained on a dataset containing 8,530 longitudinal features. To interpret the importance of each feature, we used the Explainer method from the Shapley library (v.0.42.1) with default parameters. The Shapley values^37^ were determined by averaging over randomly selected test samples, representing approximately 6.5% of the entire test dataset. To present the results, we expressed Shapley values for each feature as the mean absolute change in predicted mortality probability from an individual-specific Shapley baseline, where no longitudinal features were included.

### Statistics and reproducibility

Statistical significance was tested for algorithm performance (AUC) using permutation testing by randomly permuting group labels 1,000 times. All statistical tests were two-sided and *P* < 0.05 was considered statistically significant. No statistical methods were used to predetermine sample size because the entire Finnish population was used for the analyses, with only individuals who died, emigrated or never interacted with healthcare before the predictive intervals (which differed for the training, validation and testing datasets).

### Reporting summary

Further information on research design is available in the Nature Portfolio Reporting Summary linked to this article.

### Supplementary information

Supplementary Information Supplementary Methods, Fig. 1 and Tables 1–4.

Reporting Summary

Supplementary Table 5 TRIPOD adherence assessment form.

### Source data

Source Data Fig. 2 Statistical source data.

Source Data Fig. 3 Statistical source data.

Source Data Fig. 4 Statistical source data.

Source Data Fig. 5 Statistical source data.

Source Data Fig. 6 Statistical source data.

Source Data Extended Data Fig. 1 Statistical source data.

Source Data Extended Data Fig. 2 Statistical source data.

Source Data Extended Data Fig. 3 Statistical source data.

Source Data Extended Data Fig. 5 Statistical source data.

## Figures and Tables

**Extended Data Fig. 4:**
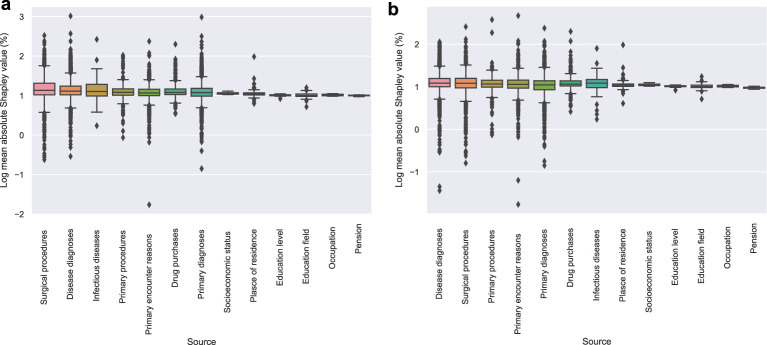
Predictor importance using Shapley values, box-and-whisker plots. **a**, Box-and-whisker plot for mean absolute Shapley values as a percentage change from the baseline by feature category. **b**, Mean absolute Shapley values excluding file last years before the predictive period. The center line of each box indicates the median, the bounds of the box represent the interquartile range (IQR, 25th to 75th percentiles), and the whiskers extend to the minima and maxima values within 1.5 times the IQR from the quartiles. Points outside this range are considered outliers and are shown as individual points

## Data Availability

Data dictionaries for the FinRegistry are publicly available on the FinRegistry website (www.finregistry.fi/finnish-registry-data). Access to the FinRegistry data can be obtained by submitting a data transfer application for individual-level data to the Finnish social and health data permit authority Findata (https://asiointi.findata.fi/). The application should include information about the purpose of data use; the requested data, including the variables, definitions for the target and control groups, and external datasets to be combined with the FinRegistry data; the dates for which data is needed; and a data use plan. The requests will be evaluated on a case-by-case basis. Once approved, the data will be sent to the secure computing environment Kapseli. It can be accessed within the European Economic Area and countries with an adequacy decision from the European Commission.

## Code availability

The essential analysis code used to produce the results is available from the FinRegistry GitHub repository at https://github.com/dsgelab/RNN.
