# Supplementary material for: Deep learning-based prediction of one-year mortality in Finland is an accurate but unfair aging marker
Source: Nat Aging. 2024 Jun 24;4(7):1014–27. doi: 10.1038/s43587-024-00657-5 (PMC11257968; doi:10.1038/s43587-024-00657-5)
Supplement: Supplementary file 1 — Supplementary Methods, Fig. 1 and Tables 1–4. [file 43587_2024_657_MOESM1_ESM.pdf]

nature aging

Supplementary information https://doi.org/10.1038/s43587-024-00657-5

Deep learning-based prediction of one-year mortality in Finland is an accurate but unfair aging marker

In the format provided by the authors and unedited

SUPPLEMENTARY INFORMATION

Contents

Supplementary Methods 2
Features 2
a. Longitudinal Features........ 2
a.1 Medical Records 2
a.1.1 Predominantly Secondary Healthcare Medical Records 2
a.1.1.1 Clinical Endpoints........ 2
a.1.1.2 NOMESCO Surgical Records........ 3
a.1.2 Primary Healthcare Medical Records 3
a.1.2.1 ICD-10 Records 3
a.1.2.2 ICPC-2 Records 3
a.1.2.3 SPAT Records 3
a.2 Medication Records 3
a.3 Infectious Diseases 4
a.4 Socioeconomic Records........ 4
a.4.1 Socioeconomic Status........ 4
a.4.2 Occupation........ 4
a.4.3 Education........ 4
a.4.4 Old Age Pension 4
a.5 Place of Residence (Geographic) Information 5
b. Fixed Over Time Features........ 5
b.1. Basic Demographic and Health 5
b.2. Social Support........ 5
b.3. Birth, Relationships, and Children 6
Supplementary Table 4: Top 100 individual predictors with the highest mean absolute SHAP values. 8
Municipality indexes for Source Data tables providing sample sizes and confidence intervals for geographic plots in Fig. 5a and Extended Data Fig. 1b. 11
Supplementary References........ 12

Supplementary Methods

Features

Both longitudinal (a) and fixed over time (b) features were considered, with a preference for a longitudinal format which retains more information. Longitudinal features included medical, socioeconomic, and geographic records, while fixed over time features included various information predominantly about demographics and health (Figure 1C).

a. Longitudinal Features

Longitudinal features are represented by medical records (a.1) which can be roughly grouped into predominantly secondary healthcare (a.1.1) and primary healthcare records (a.1.2). Predominantly secondary healthcare data can be classified in curated clinical endpoints (a.1.1.1) and surgical records (a.1.1.2). Primary healthcare records can be classified as ICD-10 records (a.1.2.1), ICPC-2 records (a.1.2.2) and SPAT records (a.1.2.3)

Medication (ATC) records (a.2) capture longitudinal medication purchases. Additional longitudinal data include infectious diseases information (a.3) and longitudinal socioeconomic information (a.4). Socioeconomic information can be categorized into socioeconomic status (a.4.1), occupation (a.4.2), education level and field (a.4.3), and old age pension (a.4.4). Finally, we considered the place of residence (geographic) information (a.5).

a.1 Medical Records

a.1.1 Predominantly Secondary Healthcare Medical Records

Predominantly secondary healthcare medical records were obtained from in-patient and out-patient registers as opposed to primary care registers.

a.1.1.1 Clinical Endpoints

Clinical Endpoints were originally defined for the FinnGen project[1] and later adapted to use within the FinRegistry project. Endpoints were predominantly generated by combining ICD[8-10, O] records coming from Healthcare (HILMO), Causes of death, and Cancer registers. In addition, for a small proportion of endpoints Drug Purchase, Drug Reimbursement, Surgical Procedure, and Primary healthcare ICD records were used. A portion of highly correlated and redundant endpoints was not included, as well as composite endpoints which only included other endpoints but no additional information from clinical registers. Endpoints generated solely from ATC records were also not included as ATC records were used separately. In addition, we have removed endpoints occurring in less than 100 thousand samples. This resulted in 2,860 clinical endpoints used for classification. Clinical endpoints and their definitions can be explored via https://risteys.finregistry.fi/. Clinical endpoints cover the entire study period from the 1st of January 1969.

a.1.1.2 NOMESCO Surgical Records

From 1997 surgical procedure records in the HealthCare (HILMO) register were recorded using the NOMESCO classification. Records consist of three alphabetic characters (positions 1-3 of the record) and two numeric characters (positions 4-5 of the records). The first three alphabetic characters denote the functional anatomical body system group, a specific location within a system group, and the method of the procedure. The remaining two numerical characters provide a more fine-grained classification of the procedure. In our study, we have excluded the two last numerical characters as it allowed us to substantially reduce the number of records from 8470 to 2290.

a.1.2 Primary Healthcare Medical Records

Primary healthcare medical records were obtained from the Primary Healthcare Register (avoHILMO), which started in 2011.

a.1.2.1 ICD-10 Records

ICD-10 records given in the primary care register consist of three to seven hierarchically organised characters. Every record begins with an alphabetical character, which is indicative of the chapter based on a body system. Further two numerical characters broadly define a health condition, and a more refined definition can be given by the remaining characters. In this study, we have used only the first three characters of primary healthcare ICD-10 records and removed rare records which resulted in a total of 1525 records.

a.1.2.2 ICPC-2 Records

ICPC-2 records predominantly encode reasons for primary healthcare visits. The classification is compatible and evolved from ICD classification to better suit primary healthcare needs. It allows recording patients' reasons for encounters, health problems/diagnoses and primary healthcare procedures and interventions. In this study, we have used a total of 1089 records.

a.1.2.3 SPAT Records

The Finnish classification of functions in outpatient primary healthcare (SPAT) is used to describe functions and procedures in outpatient primary healthcare. In this study, we have used 361 unique 8-symbol SPAT records.

a.2 Medication Records

ATC records classify medicines by active substances in a hierarchical fashion with five different levels. ATC records were obtained from the Drug Purchases register which includes the medicines purchased via pharmacies with a doctor's prescription and does not include medicines administered during hospital admission. There were 1431 unique ATC records recorded with the register which is available for the 1995-2019 period. We, however, used only the first 5 symbols out of 7 and removed rare ATC records (<1 in 100:000) resulting in 440 unique records retained.

a.3 Infectious Diseases

The register of infectious diseases is based on disease notifications from medical doctors and laboratories. In our study, we have used information about microbes which caused an infectious disease from 1994 until the predictive interval. In total, there were 170 unique records corresponding to specific microbe groups causing infectious diseases.

a.4 Socioeconomic Records

In this study, socioeconomic information was used longitudinally as the dynamics of those variables can capture the environmental and social aspects influencing health.

a.4.1 Socioeconomic Status

Socioeconomic status was based on the information about the main activity, occupation, professional status, and industry of an individual. Eight different socioeconomic status categories (records) were used: lower-level employees, manual workers, upper-level employees, self-employed, students, pensioners, others, and unknown. For individuals for whom the data was available on average socioeconomic status changed 3.4 times. For persons aged 0-15 socioeconomic status was based on the socioeconomic status of the reference person of the household-dwelling. Socioeconomic status was obtained from Statistics Finland and was available from 1970.

a.4.2 Occupation

Occupation data was obtained from Statistics Finland and was available from the year 1995 and each occupation was coded hierarchically following the structure of ISCO - International Standard Classification of Occupations[2]. In the register, there were 1277 unique occupations recorded but for our purposes lower level of detail was sufficient and we retained only the first symbol of each record, resulting in 11 unique occupation categories: service and sales workers, professionals, technicians and associate professionals, craft and related trades workers, plant and machine operators, and assemblers, elementary occupations, clerical and support workers, skilled agricultural, forestry and fishery workers, managers, armed forces, unknown.

a.4.3 Education

Records for education level and education field were used. Education level is ordered from the lowest to the highest, ranging from secondary to doctoral level education (13 categories in total). The education field based on the International Standard Classification of Education (ISCED) classification contains hierarchical 3-4 symbol records signifying the education area from the broad (first symbol) to the more detailed (further symbols). In total 100 unique education-level records were used. Education information was obtained from Statistics Finland and was available from 1970.

a.4.4 Old Age Pension

Earnings-related old-age pension amounts were taken from the Finnish Centre for Pensions register spanning 1990-2020. The pension amount variable was discretized into a

categorical variable with 20 contiguous levels, each having an equal number of samples. It was used longitudinally at each year the pension was received by an individual.

a.5 Place of Residence (Geographic) Information

Finland has 309 municipalities (2021) and in this study, the geographical location of the individuals was based on the municipality. The geographical information was available from 1964 onwards and to account for changes in municipality definitions (area changes, new municipalities appearing and old disappearing) throughout this follow-up period, the location information was harmonised based on 309 municipalities as defined in 2021. In the models, this information was used longitudinally as individuals for whom the data was available changed the municipality in which they lived on average 2.1 times. As geographic information was available only for index individuals, for individuals born on and after 2010 their mothers' geographic information was used. Living information was obtained from the DVV register.

b. Fixed Over Time Features

Fixed over time features were grouped into basic demographic and health (b.1), social support (b.2) and birth, relationships, and children (b.3).

b.1. Basic Demographic and Health

Basic demographic features predominantly come from DVV registers. However, here we also included basic health features. Number of drug purchases and drug prescriptions from Kela and Kanta registers. Smoking status was recorded in Avohilmo and Birth registers and was available for 30% of the study population (see Supplementary Table 1).

***Supplementary Table 1*: Basic demographic and health fixed over time features.**

Feature description	Source	Type
Age in days	DVV	Continuous
Sex	DVV Relatives	Binary
Index person (living in Finland on 01/01/2010)	DVV	Binary
Number of children	DVV Relatives	Ordinal
Record(s) in THL Social assistance reg.	THL Soc. Assist.	Binary
Record(s) in THL Social Hilmo reg.	THL Soc. Hilmo	Binary
Record(s) in THL Infectious diseases reg.	THL Infect. dis.	Binary
Record(s) in THL malformations reg.	THL Malformations	Binary
Record(s) in THL cancer reg.	THL Cancer	Binary
Latest recorded smoking status	THL AvoHilmo + Birth	6 categories
Mother tongue (Fi. / Sw. / Ru. / other / unknown)	DVV Relatives	5 categories

b.2. Social Support

We included here information from the THL Register of social assistance about monetary support received by individuals due to lack or insufficiency of income. In addition, here we have included information about the duration of institutional care received by individuals (see Supplementary Table 2).

***Supplementary Table 2*: Social support fixed over time features.**

Feature description	Source	Type
Total amount of received social assistance	THL Soc. Assist.	Continuous
Duration of social assistance in moths	THL Soc. Assist.	Continuous
How many years social assistance spanned	THL Soc. Assist.	Continuous
Duration in institutional care	THL Soc. Hilmo	Continuous

b.3. Birth, Relationships, and Children

THL Birth register has a vast amount of information both about newborns and their mothers. Birth information and complications for mothers are recorded in the Hilmo register, however, in that register, there is no such information recorded for newborns. Therefore, here we have included features about a person and their mother at the time of birth and immediately after. Here we also used information from the THL malformation register signifying the severity of malformation and also some features about relationships occurring much later in life (see Supplementary Table 3).

***Supplementary Table 3*: Birth, relationships, and children fixed over time features.**

Feature description	Source	Type
Mother's age at birth	THL Birth	Continuous
Best estimate of pregnancy duration in days	THL Birth	Continuous
Maternal marital status	THL Birth	9 categories
Maternal cohabiting relationship	THL Birth	3 categories
Previous pregnancies	THL Birth	Ordinal
Previous miscarriages	THL Birth	Ordinal
Previous induced abortions	THL Birth	Ordinal
Previous ectopic pregnancies	THL Birth	Ordinal
Previous births	THL Birth	Ordinal
Previous stillborn births	THL Birth	Ordinal
Total number of check-ups	THL Birth	Ordinal
Number of outpatient check-ups	THL Birth	Ordinal
Mothers smoking status at birth	THL Birth	5 categories
The way in which the child was born	THL Birth	9 categories
Number of foetuses, 1-3	THL Birth	Ordinal
Birth weight, g	THL Birth	Continuous
Birth length, cm	THL Birth	Continuous
New-born assessment at 7d	THL Birth	6 categories
Score of a Apgar test given to newborns at 1 min	THL Birth	Ordinal
Score of a Apgar test given to newborns at 5 min	THL Birth	Ordinal
Glucose tested and pathological	THL Birth	Binary
Artificial insemination (IVF, ICSI, FET)	THL Birth	Binary
Resuscitated immediately after birth	THL Birth	Binary
The new-born is resuscitated by the age of 7d	THL Birth	Binary
Severity of birth malformation	THL Malformations	7 categories
Ever married	DVV Marriage	3 categories
Ever divorced	DVV Marriage	3 categories
Recorded in birth registry as a mother	THL Birth	Binary
Recorded in birth registry as a child	THL Birth	Binary

Supplementary Table 4: Top 100 individual predictors with the highest mean absolute SHAP values.

No.	Abs. SHAP	Source	Code	Code name
1	68.8	Surgical procedures	WD1	Widespread tumour treatments
2	55.1	Secondary diagnoses	G6_NEUATR	Systemic atrophies primarily affecting the central nervous system
3	52.2	Surgical procedures	SCA	Preventive oral health care
4	48.0	Secondary diagnoses	K11_DEPOSITS	Deposits [accretions] on teeth
5	47.5	Surgical procedures	TK8	Haemodialysis
6	47.3	Primary diagnoses	K70	Alcoholic liver disease
7	47.1	Secondary diagnoses	K11_ALCOLIV	Alcoholic liver disease
8	46.4	Drug purchases	B03XA	Other antianemic preparations
9	46.0	Drug purchases	L01XE	Protein kinase inhibitors
10	45.6	Surgical procedures	JDB	Gastric ostomy
11	45.4	Surgical procedures	IAZ	Psychiatric consultation
12	44.6	Surgical procedures	IEP	NA
13	44.5	Drug purchases	L01BB	Purine analogues
14	43.9	Surgical procedures	RL1	NA
15	43.1	Secondary diagnoses	F5_OTHRET	Other and unspecified mental retardation
16	43.0	Surgical procedures	QB9	Photography of skin or subcutaneous tissue
17	42.8	Secondary diagnoses	K11_POSTOPDIS	Postprocedural disorders of digestive system, not elsewhere classified
18	42.2	Drug purchases	A04AA	Serotonin (5-HT3) antagonists
19	41.8	Secondary diagnoses	Q17_CONGEMALFO_RESPSYSTEM	Congenital malformations of the respiratory system
20	41.7	Secondary diagnoses	CHIRHEP_NAS	Cirrhosis of liver, NAS
21	41.5	Surgical procedures	WD3	Widespread tumour treatments
22	40.8	Secondary diagnoses	Q17_CYSTIKIDNEY_DISEA	Cystic kidney disease
23	38.5	Secondary diagnoses	P16_BIRTH_ASPHYXIA	Birth asphyxia
24	38.3	Drug purchases	A12AA	Calcium
25	38.1	Surgical procedures	XX4	Canalization
26	36.6	Secondary diagnoses	K11_TEETH_HARD	Other diseases of hard tissues of teeth
27	36.5	Secondary diagnoses	D3_COAGDEF	Chronic Coagulation defects
28	36.2	Surgical procedures	GBB	Procedures related to a tracheal ring
29	36.0	Drug purchases	A11CC	Vitamin D and analogues
30	35.7	Drug purchases	V03AE	Drugs for treatment of hyperkalaemia and hyperphosphatemia
31	35.2	Secondary diagnoses	E4_DM1REN	Type 1 diabetes with renal complications
32	35.0	Drug purchases	N04BX	Other dopaminergic agents
33	34.9	Secondary diagnoses	INV_VENT	Invasive ventilation
34	34.7	Surgical procedures	JA3	Channelling of the abdominal cavity
35	34.5	Surgical procedures	FM2	Myocardial perfusion procedures
36	34.4	Surgical procedures	ZYB	NA
37	34.4	Surgical procedures	YA1	Head and neck procedures
38	34.1	Secondary diagnoses	DM_NEPHROPATHY	Diabetic nephropathy
39	34.0	Surgical procedures	TJD	Placement of a nasogastric or nasogastric duodenal tube

40	34.0	Secondary diagnoses	Q17_OTHER_SPECIFE_CONGEMALFO_SYNDR_AFFECTING_MULTIPLE_SYSTEMS	Other specified congenital malformation syndromes affecting multiple systems
41	33.9	Surgical procedures	WD2	Widespread tumour treatments
42	33.8	Surgical procedures	IBZ	Psychiatric evaluation
43	33.8	Secondary diagnoses	Q17_CONGEMALFO_URINARY_SYSTEM	Congenital malformations of the urinary system
44	33.6	Secondary diagnoses	G6_HCOBSTR	Obstructive hydrocephalus
45	33.5	Secondary diagnoses	C3_LUNG_NONSMALL	Non-small cell lung cancer
46	33.4	Secondary diagnoses	KRA_PSY_MENTALRET	Mental retardation
47	33.4	Surgical procedures	IGP	NA
48	33.4	Secondary diagnoses	P16_OTH_DISORD_ORIGINA_PERINA_PERIOD	Other disorders originating in the perinatal period
49	33.3	Surgical procedures	IGV	NA
50	33.3	Secondary diagnoses	I9_VARICVEOES	Oesophageal varices
51	33.2	Secondary diagnoses	KRA_PSY_SUBSTANCE	Substance abuse
52	33.1	Secondary diagnoses	K11_HEPFAIL	Hepatic failure, not elsewhere classified
53	33.1	Secondary diagnoses	Q17_CONGEMALFO_NERVOUS_SYSTEM	Congenital malformations of the nervous system
54	33.0	Secondary diagnoses	F5_MODRET	Moderate mental retardation
55	32.9	Secondary diagnoses	E4_DM2REN	Type 2 diabetes with renal complications
56	32.8	Surgical procedures	WD4	Antibody therapy of metastasized malignancy
57	32.8	Secondary diagnoses	F5_OPIOIDS	Mental and behavioural disorders due to opioids
58	32.5	Surgical procedures	RT2	NA
59	32.4	Secondary diagnoses	CD2_MULTIPLE_MYELOMA_PLASMA_CELL	Multiple myeloma and malignant plasma cell neoplasms
60	32.3	Surgical procedures	R14	Drawing up a rehabilitation plan
61	32.2	Surgical procedures	WW5	Blood transfusion
62	32.1	Surgical procedures	1XC	Treatment with monoclonal antibodies
63	32.0	Secondary diagnoses	D3_COAGDEFNAS	Other and unspecified coagulation defects
64	31.6	Secondary diagnoses	L12_DECUBITANSULCERANDPRESSURE	Decubitus ulcer and pressure area
65	31.6	Secondary diagnoses	K11_GINGIVA	Other disorders of gingiva or/and edentulous alveolar ridge
66	30.6	Encounter reasons	N99	Neurological disease other
67	30.6	Encounter reasons	P85	Developmental disability
68	30.2	Surgical procedures	RT4	NA
69	30.2	Surgical procedures	ZYC	Remote contact
70	30.1	Surgical procedures	WB1	Multiple cytostatic treatment of a tumour
71	30.1	Secondary diagnoses	CD2_LYMPHOID_LEUKAEMIA	Lymphoid leukaemia
72	30.1	Secondary diagnoses	N14_CHRONTUBULOINTNEPHRITIS	Chronic tubulo-interstitial nephritis
73	29.9	Surgical procedures	3AC	NA
74	29.9	Primary diagnoses	G35	Multiple sclerosis
75	29.9	Surgical procedures	RT1	Rheumatoid arthritis procedures
76	29.8	Surgical procedures	IBA	Psychiatric evaluation

77	29.8	Secondary diagnoses	DM_POLYNEURO	Diabetic polyneuropathy
78	29.6	Drug purchases	L04AD	Calcineurin inhibitors
79	29.6	Surgical procedures	ICZ	Preparation of psychiatric treatment plan
80	29.5	Surgical procedures	IGY	Psychiatric treatment contact
81	29.5	Surgical procedures	JF1	Small intestine examinations
82	29.3	Surgical procedures	JN4	Extensive body imaging
83	29.3	Secondary diagnoses	DRUGADVERS_NEUTROPENIA	Drug-induced neutropenia
84	28.9	Secondary diagnoses	J10_RESPOTHER	Other diseases of the respiratory system
85	28.6	Surgical procedures	XW0	Bone marrow sampling
86	28.5	Surgical procedures	ICB	Psychiatric treatment plan
87	28.4	Secondary diagnoses	G6_HCOTHUNS	Other and unspecified hydrocephalus
88	28.4	Secondary diagnoses	F5_CANNABIS	Mental and behavioural disorders due to cannabinoids
89	28.1	Secondary diagnoses	N14_CHRONKIDNEYDIS	Chronic kidney disease
90	28.1	Secondary diagnoses	G6_ALCODEGEN	Degeneration of the brain due to alcohol
91	28.1	Surgical procedures	TPX	Establishing a permanent injection route
92	28.1	Secondary diagnoses	F5_OTHERSUB	Mental and behavioural disorders due to multiple drug use and use of multiple drugs and use of other psychoactive substances
93	28.1	Surgical procedures	GA1	Ultrasound examination of the thorax
94	28.0	Secondary diagnoses	F5_KELARET	Kela-code for behavioural disturbances in mental retardation
95	28.0	Secondary diagnoses	G6_CP	Cerebral palsy
96	28.0	Secondary diagnoses	H7_RETINOPATHYDIAB_PROLIF	Proliferative diabetic retinopathy
97	28.0	Secondary diagnoses	N14_ACUTERENFAIL	Acute renal failure
98	27.9	Secondary diagnoses	ALCOPANCCHRON	Alcohol-induced chronic pancreatitis
99	27.9	Secondary diagnoses	K11_CHRONPANC	Chronic pancreatitis
100	27.8	Secondary diagnoses	F5_PSYCHDEVOTH	Other and unspecified disorders of psychological development

Municipality indexes for Source Data tables providing sample sizes and confidence intervals for geographic plots in Fig. 5a and Extended Data Fig. 1b.

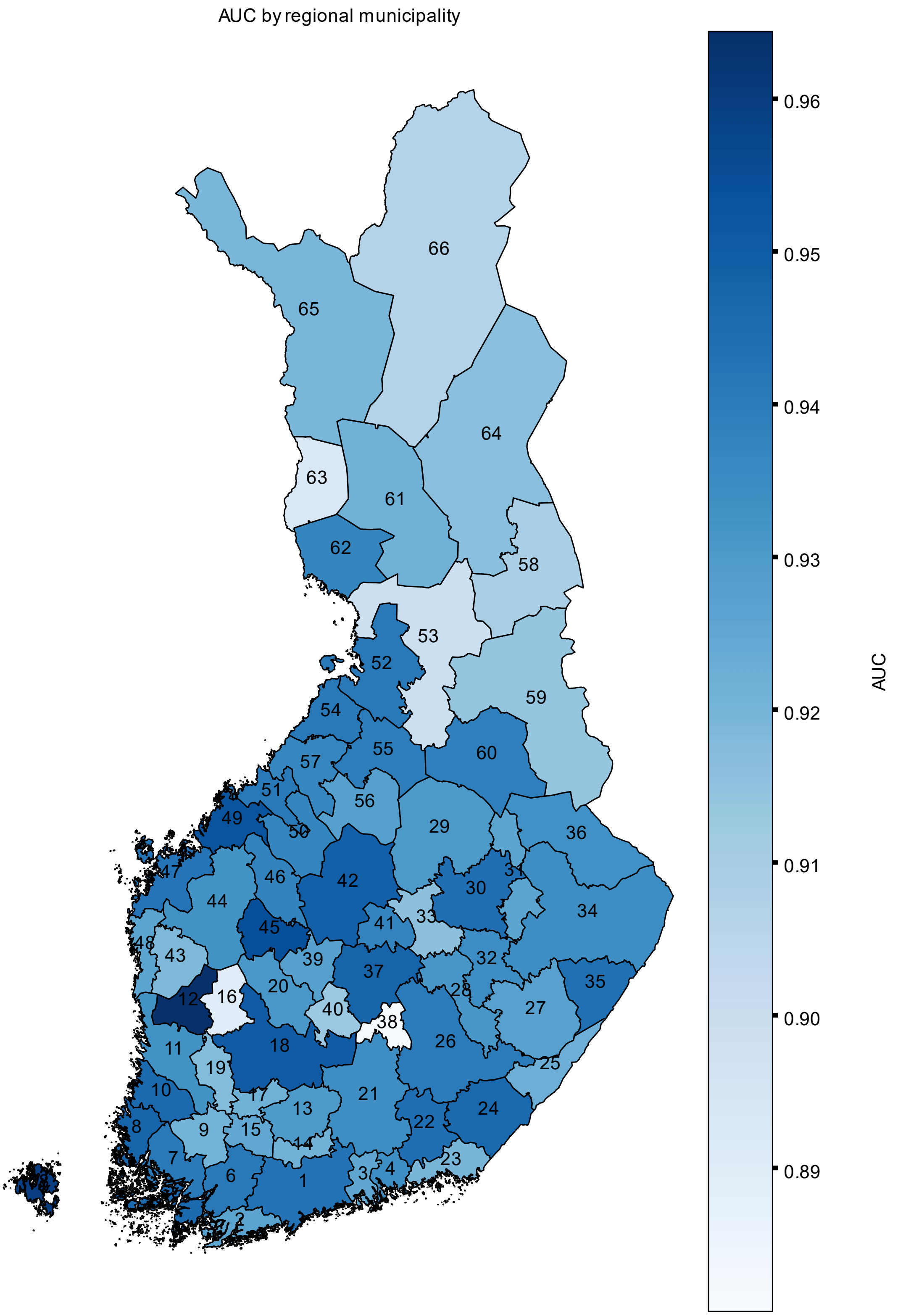

Supplementary Fig. 1: AUC variation by a regional municipality in Finland with municipality index which corresponds with an index in Source Data file providing information about sample size, number of cases, AUC, and confidence intervals for each regional municipality.

Supplementary References

1. Kurki, M. I. *et al.* FinnGen provides genetic insights from a well-phenotyped isolated population. *Nature* **613**, 508–518 (2023).
2. International Labour Organization. International Standard Classification of Occupations (ISCO-08): Structure, group definitions and correspondence tables. https://www.ilo.org/public/english/bureau/stat/isco/ (2012).
