# Supplementary material for: Deep learning-based prediction of one-year mortality in Finland is an accurate but unfair aging marker
Source: Nat Aging. 2024 Jun 24;4(7):1014–27. doi: 10.1038/s43587-024-00657-5 (PMC11257968; doi:10.1038/s43587-024-00657-5)
Supplement: Supplementary file 2 — Reporting Summary [file 43587_2024_657_MOESM2_ESM.pdf]

nature portfolio

Corresponding author(s): Andrea Ganna

Last updated by author(s): Feb 12, 2023

Reporting Summary

Nature Portfolio wishes to improve the reproducibility of the work that we publish. This form provides structure for consistency and transparency in reporting. For further information on Nature Portfolio policies, see our Editorial Policies and the Editorial Policy Checklist.

Statistics

For all statistical analyses, confirm that the following items are present in the figure legend, table legend, main text, or Methods section.

n/a	Confirmed	
☐	☒	The exact sample size (*n*) for each experimental group/condition, given as a discrete number and unit of measurement
☐	☒	A statement on whether measurements were taken from distinct samples or whether the same sample was measured repeatedly
☐	☒	The statistical test(s) used AND whether they are one- or two-sided<br>*Only common tests should be described solely by name; describe more complex techniques in the Methods section.*
☐	☒	A description of all covariates tested
☐	☒	A description of any assumptions or corrections, such as tests of normality and adjustment for multiple comparisons
☐	☒	A full description of the statistical parameters including central tendency (e.g. means) or other basic estimates (e.g. regression coefficient) AND variation (e.g. standard deviation) or associated estimates of uncertainty (e.g. confidence intervals)
☐	☒	For null hypothesis testing, the test statistic (e.g. *F*, *t*, *r*) with confidence intervals, effect sizes, degrees of freedom and *P* value noted<br>*Give P values as exact values whenever suitable.*
☐	☒	For Bayesian analysis, information on the choice of priors and Markov chain Monte Carlo settings
☐	☒	For hierarchical and complex designs, identification of the appropriate level for tests and full reporting of outcomes
☒	☐	Estimates of effect sizes (e.g. Cohen's *d*, Pearson's *r*), indicating how they were calculated

Our web collection on statistics for biologists contains articles on many of the points above.

Software and code

Policy information about availability of computer code

Data collection	N/A
Data analysis	For analyses python 3.9.1 was used with pytorch 1.7.1 and py-xgboost 0.90 for classification, optuna 2.10.1 for parameter optimization and other standard data science libraries including scikit-learn 0.23.2, numpy 1.19.2, pandas 1.2.3, scipy 1.6.0, matplotlib 3.3.2. Essential analysis code used to produce the results is available in the FinRegistry GitHub at: https://github.com/dsgelab/RNN.

For manuscripts utilizing custom algorithms or software that are central to the research but not yet described in published literature, software must be made available to editors and reviewers. We strongly encourage code deposition in a community repository (e.g. GitHub). See the Nature Portfolio guidelines for submitting code & software for further information.

Data

Policy information about availability of data

All manuscripts must include a data availability statement. This statement should provide the following information, where applicable:

- Accession codes, unique identifiers, or web links for publicly available datasets
- A description of any restrictions on data availability
- For clinical datasets or third party data, please ensure that the statement adheres to our policy

Data dictionaries for FinRegistry are publicly available on the FinRegistry website (www.finregistry.fi/finnish-registry-data). Access to FinRegistry data can be obtained by submitting a data permit application for individual-level data for the Finnish social and health data permit authority Findata (https://asiointi.findata.fi/).

The application includes information on the purpose of data use; the requested data, including the variables, definitions for the target and control groups, and external datasets to be combined with FinRegistry data; the dates of the data needed; and a data utilization plan. The requests are evaluated on a case-by-case basis. Once approved, the data are sent to a secure computing environment Kapseli and can be accessed within the European Economic Area (EEA) and countries with an adequacy decision from the European Commission.

Research involving human participants, their data, or biological material

Policy information about studies with human participants or human data. See also policy information about sex, gender (identity/presentation), and sexual orientation and race, ethnicity and racism.

Reporting on sex and gender	Sex was reported as it was recorded in official Digital, and Population Data Services Agency (Finland, https://dvv.fi/en/individuals/) registers. We only used terms female/male and sex.
Reporting on race, ethnicity, or other socially relevant groupings	Imigration status and marital status were used to group individuals and data for grouping came from official Digital, and Population Data Services Agency (Finland, https://dvv.fi/en/individuals/) living history and marriage registers. Grouping indivuals into having ant not having mental health diagnoses was based on Hilmo registers and grouping into groups of different pension size was based on Statistics Finland Pension register.
Population characteristics	It was a population representative sample using data from a full population of a country.
Recruitment	Register data was used, which did not involve participant recruitment.
Ethics oversight	FinRegistry is a collaboration project of the Finnish Institute for Health and Welfare (THL) and the Data Science Genetic Epidemiology research group at the Institute for Molecular Medicine Finland (FIMM), University of Helsinki. The FinRegistry project has received the following approvals for data access from the National Institute of Health and Welfare (THL/1776/6.02.00/2019 and subsequent amendments), DVV (VRK/5722/2019-2), Finnish Center for Pension (ETK/SUTI 22003) and Statistics Finland (TK-53-1451-19). The FinRegistry project has received IRB approval from the National Institute of Health and Welfare (Kokous 7/2019).

Note that full information on the approval of the study protocol must also be provided in the manuscript.

Field-specific reporting

Please select the one below that is the best fit for your research. If you are not sure, read the appropriate sections before making your selection.

☒ Life sciences ☐ Behavioural & social sciences ☐ Ecological, evolutionary & environmental sciences

For a reference copy of the document with all sections, see nature.com/documents/nr-reporting-summary-flat.pdf

Life sciences study design

All studies must disclose on these points even when the disclosure is negative.

Sample size	No statistical methods were used to predetermine the sample size as the entire Finnish population was used for analyses with only individuals who have died, emigrated, or have never interacted with healthcare before predictive intervals (which differed for training, validation, and testing sets).
Data exclusions	No data was excluded from analyses, except in an above "Sample size" section described exclusions.
Replication	For our study, we have used the data for an entire Finnish population. To test result generalizability we have used temporal approach (validation and test sets were shifted forward in time making the data used for testing "unseen" during training) and compared prediction performance within different regions of Finland. The results can be replicated in other countries with similar register data availability (Nordic countries), but to our knowledge, they have not been replicated yet.
Randomization	Training, Testing and Validation subsamples were selected randomly.
Blinding	Randomization and Blinding were not very relevant as in this machine learning study there was no traditionally defined experimental/control groups. Other groupings (e.g. labels) were defined by events in individuals registry history (e.g. death)

Reporting for specific materials, systems and methods

We require information from authors about some types of materials, experimental systems and methods used in many studies. Here, indicate whether each material, system or method listed is relevant to your study. If you are not sure if a list item applies to your research, read the appropriate section before selecting a response.

Materials & experimental systems

n/a	Involved in the study
☒	☐ Antibodies
☒	☐ Eukaryotic cell lines
☒	☐ Palaeontology and archaeology
☒	☐ Animals and other organisms
☒	☐ Clinical data
☒	☐ Dual use research of concern
☒	☐ Plants

Methods

n/a	Involved in the study
☒	☐ ChIP-seq
☒	☐ Flow cytometry
☒	☐ MRI-based neuroimaging
